# Evaluation of the Data Quality from a Round-Robin Test of Hyperspectral Imaging Systems

**DOI:** 10.3390/s20143812

**Published:** 2020-07-08

**Authors:** Ruven Pillay, Marcello Picollo, Jon Yngve Hardeberg, Sony George

**Affiliations:** 1 NTNU-Norwegian University of Science and Technology, Department of Computer Science, Teknologivegen 22, N-2815 Gjøvik, Norway; jon.hardeberg@ntnu.no (J.Y.H.); sony.george@ntnu.no (S.G.); 2 C2RMF-Centre de Restauration et Recherche des Musées de France, Porte des Lions-Palais du Louvre, 75001 Paris, France; 3 IFAC-CNR-Istituto di Fisica Applicata “Nello Carrara” del Consiglio Nazionale delle Ricerche, Via Madonna del Piano 10, 50019 Firenze, Italy; m.picollo@ifac.cnr.it

**Keywords:** round-robin test, hyperspectral imaging, spectral quality, data quality, spectroscopy, colorimetry

## Abstract

In this study, the results from a round-robin test of hyperspectral imaging systems are presented and analyzed. Fourteen different pushbroom hyperspectral systems from eight different institutions were used to acquire spectral cubes from the visible, near infra-red and short-wave infra-red regions. Each system was used to acquire a common set of targets under their normal operating conditions with the data calibrated and processed using the standard processing pipeline for each system. The test targets consisted of a spectral wavelength standard and of a custom-made pigment panel featuring Renaissance-era pigments frequently found in paintings from that period. The quality and accuracy of the resulting data was assessed with quantitative analyses of the spectral, spatial and colorimetric accuracy of the data. The results provide a valuable insight into the accuracy, reproducibility and precision of hyperspectral imaging equipment when used under routine operating conditions. The distribution and type of error found within the data can provide useful information on the fundamental and practical limits of such equipment when used for applications such as spectral classification, change detection, colorimetry and others.

## 1. Introduction

Hyperspectral imaging provides a powerful combination of high spectral resolution and dense spatial mapping and has become a valuable analytical tool in a wide range of fields, including remote sensing [1], food science [2], astronomy [3], mineralogy [4], agriculture [5], medicine [6], the study of art [7] and many others. Hyperspectral data can be used in a number of ways, including, for example, materials mapping and identification, the detection of hidden features, change monitoring and many other applications.

However, the accuracy of real-world hyperspectral imaging systems is not only limited by the technical characteristics of the hyperspectral cameras themselves, but also by the overall setup, how they are used and how the acquired data is processed. These factors include the power and spectral content of the lighting used, the optical geometry, the integration time and acquisition parameters, as well as the kind of processing and calibration of the data that is carried out. The reproducability of hyperspectral data between systems, institutions and indeed over time is an important consideration in many applications, especially when the comparison of data is important. For example, when monitoring change, when performing classification using 3rd party spectral libraries or when combining a variety of data sets in a multimodal analysis.

In order, therefore, to assess the real-world reproducability and limits of hyperspectral imaging, we will in this study compare a number of different hyperspectral data sets acquired of a single set of targets, but using different hyperspectral imaging equipment from different institutions.

The experimental data for this study was acquired as part of a round-robin test for the COSCH project [8] involving nineteen different institutions, which included research laboratories, universities, equipment manufacturers and museums. The goal was to evaluate the effective limits in accuracy of the spectral imaging equipment currently in use within these institutions. The data acquisition was carried out using either multispectral or hyperspectral equipment from different manufacturers and with different experimental acquisition setups, procedures and methodologies. The aim of the comparison was *not* to compare the hardware specifications or raw performance of the imaging devices themselves, but to measure the resulting effective performances of the systems globally under their standard operating conditions and after the application of the calibration and processing workflows that are usually applied by each institution to their system. The variability due to the different setups, operating procedures and the way data was processed was, therefore, an important factor to take account of and include in the study. In this way, an insight into the practical limits in accuracy of hyperspectral systems within routine operating environments could be gleaned.

Round-robin tests are a useful means of comparing equipment or methodologies and have been successfully carried out in various related fields including the measurement of the BRDF (Bidirectional Reflectance Distribution Function) of diffuse reflectors [9], for radiometric calibration of a satellite multispectral sensor [10] or in laboratory settings for field spectrometers [11]. Although the round-robin test was mainly concerned with the study of cultural heritage, the methodology and results from the test presented here are generic and relevant to a wide range of fields.

The round-robin test included both multispectral as well as hyperspectral imaging equipment. However, for this study, we have limited ourselves to a subset of the participants who were able to provide data from pushbroom hyperspectral imaging devices. This subset of data was obtained from eight participating institutions including cultural heritage conservation centers, specialist research laboratories, research libraries, universities as well as data obtained directly from two different hyperspectral camera manufacturers.

These participants all used broadly similar pushbroom hyperspectral imaging devices, configured to use either horizontal or vertical linear translation stages for scanning. For all systems, the light sources moved together with the camera, providing constant, though not necessarily uniform, illumination. Each hyperspectral camera system was used to acquire the set of targets under their usual operating conditions. Calibration and processing of the data was carried out according to the procedures that were routine for that particular system and institution. The data presented here includes data from the visible and near infra-red (VNIR) spectral region (approximately 400–1000 nm) as well as from the short-wave infra-red (SWIR) spectral region (approximately 1000–2500 nm). Hyperspectral cameras with different detectors were used for the VNIR and SWIR regions, with Silicon-based CCD (Charged Couple Device) or CMOS (Complementary Metal Oxide Semiconductor) detectors for the VNIR and for the SWIR either InGaAs (Indium Gallium Arsenide) based detectors with spectral ranges of approximately 1000–1700 nm or MCT (Mercury Cadmium Telluride) detectors with spectral ranges of approximately 1000–2500 nm.

All eight institutions were able to provide usable data in the VNIR region with 6 of these also providing data in the SWIR region. The systems included 8 different VNIR cameras from 3 different manufacturers and 6 different SWIR cameras from 2 different manufacturers. The essential spectral and spatial characteristics of the acquired data are given in Table 1, which show the hardware used, the spectral ranges, the number of bands captured and the spatial resolution at which the data was acquired. More detailed hardware characteristics and acquisition parameters used can be found in the Appendix B in Table A2.

Each hyperspectral system was a production-ready system which had been characterized and “factory-calibrated” for spectral alignment either directly by the manufacturer or by in-house specialists within the institution. As we can see from Table 1, although the hardware used was often very similar or even identical, each system was configured to operate differently with different spectral ranges, different numbers of spectral bands, different average bandwidths and different spatial resolutions.

In addition, each system possessed slightly different acquisition optical geometries; different illuminant light sources; different acquisition times and differed as to whether techniques such as binning and averaging were used etc. There was also variability in the way raw data was processed by each institution. In all cases, radiometric calibration was carried out, which entailed the use of a known traceable reflectance standard (typically Spectralon^®^, manufactured by Labsphere Inc.). Although there were variations in the sophistication of the calibration procedures used by each institution, the basic principles used by all groups were identical, consisting essentially of subtraction of the dark noise current and then signal normalization to “absolute” reflectance. In all cases, this involved the acquisition of a dark current signal using an identical integration time to the scan, which was averaged over 10–100’s of acquisitions. And for normalization, this involved scanning a reflectance standard and a uniform neutral target large enough to fill the entire field of view in order to correct for inhomogeneity in the illumination and to scale the pixel responses to absolute reflectance factor. Several systems performed this in a single step with a reflectance standard large enough to fill the field of view. Several systems additionally had accurate pixel-wise relative responsivity values generated using an integrating sphere carried out in-house or from a manufacturer-supplied characterization. The calibration routines were, therefore, similar, but not identical and followed to varying degrees the hyperspectral acquisition, processing and calibration workflows described in Reference [12]. Although differences in the resulting spectra in such circumstances are to be expected, the scale of this variability provides important information on the ability to compare data across systems and institutions.

The results presented here are from two of the test targets used for the round-robin test: a diffuse lambertian Zenith Polymer^®^ wavelength standard and a custom-made pigment panel made up of 7 different historical pigments in a tempera binder. For an overview of the round-robin test, see Reference [13]. Details and results from the other test targets used in the round-robin test (a Macbeth ColorChecker chart and a 19th Century Russian icon) can be found in References [14] and [15] respectively.

To evaluate the data, a number of different analyses were carried out on the data to ascertain the accuracy of the data, the types of errors within the data and the variability between the different sets of data. These analyses included quantitative evaluations of the accuracy of the data in terms of spectral accuracy, geometric accuracy and colorimetric accuracy. In addition, the quality of the data in terms of noise was also quantified and compared between data sets.

## 2. Test Targets and Methodology

The two test targets used for this analysis were a wavelength standard and a custom-made pigment panel made up of historical pigments in an egg tempera binder.

### 2.1. Wavelength Standard

**Figure 1:**
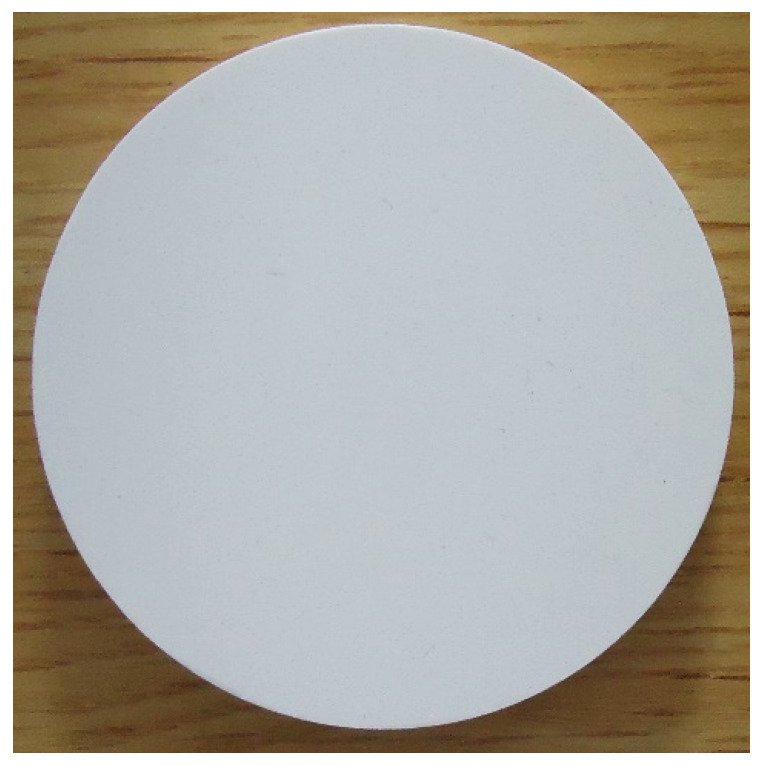
Rare-Earth Doped Wavelength Standard.

Wavelengths standards are reflectance targets designed for precise wavelength calibration of spectrophotometers, reflectometers and other spectral instruments. The standard used in this study was a Zenith Polymer^®^ wavelength standard (manufactured by SphereOptics GmbH) (Figure 1), which is a chemically inert diffuse lambertian reflectance standard composed of PTFE (Polytetrafluoroethylene) doped with the oxides of the rare earth elements Holmium, Erbium and Dysprosium. This combination gives the standard a stable spectrum of characteristic, well-defined and narrow features over the UV, visible and near infra-red spectral ranges, which is suited for use in accurate spectral calibration. The standard is supplied with traceable, laboratory-certified reference reflectance measurements covering the entire spectral range.

### 2.2. Pigment Panel

**Figure 2:**
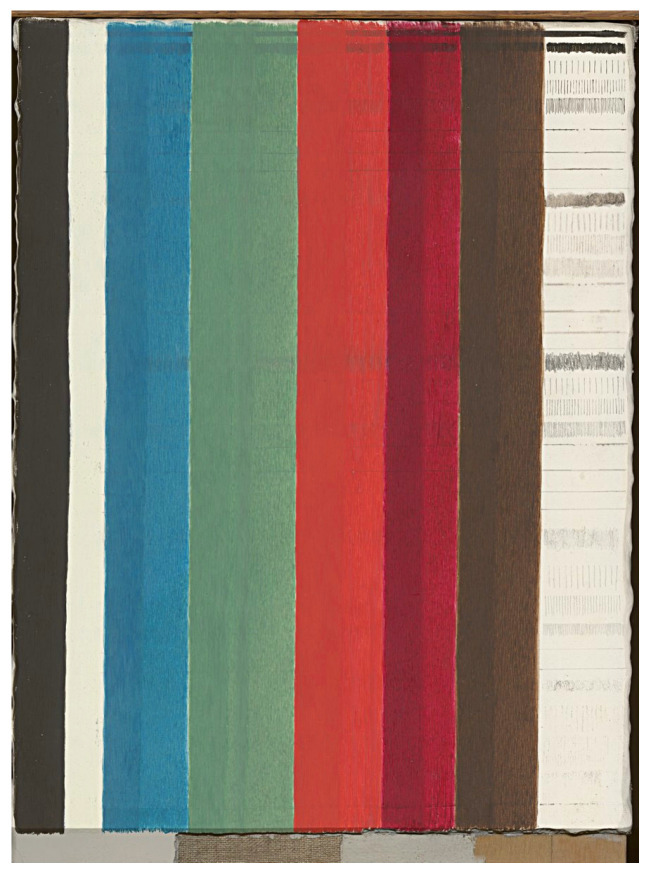
Pigment panel with vertical strips of historical pigments mixed in an egg tempera binder. From left to right: ivory black, lead white, azurite, malachite, vermilion, carmine and burnt umber. To the right of the pigments is an unpainted gypsum ground layer with various types of metalpoint drawing.

The other test target evaluated in this study was a painted panel of approximately 22 × 29 cm that was made up of historical pigments at different concentrations in an egg tempera binder. The panel, shown in Figure 2, was created especially for the round-robin test by IFAC-CNR, one of the participating institutions. The paint preparation and application aimed to authentically reproduce the medieval Tuscan painting technique described in Cennino Cennini’s 15th century *Il Libro dell’Arte* [16].

The panel consists of a wooden support with a gypsum ground, a canvas layer, and a second gypsum ground layer. Before application of the paint layer, five types of drawing materials (watercolor, charcoal, graphite, a lead and tin-based metalpoint and a lead-based metalpoint) were used to create lines and line patterns that were then covered with paints applied with two different thicknesses.

Seven pigments (obtained from Zecchi - Colori, Belle Arti, Florence) were chosen which were widely used during the Renaissance and were commonly applied using an egg tempera technique, The pigments were Burnt Umber, Carmine, Vermilion, Malachite, Azurite, Lead White, and Ivory Black (the compositions of the pigments can be found in the Appendix B in Table A1). These pigments were mixed in an egg tempera binder and applied to the panel creating the vertical strips seen in Figure 2. Additional details on the panel and on the source and composition of the pigments used for the panel can be found in Reference [17].

The pigments were chosen as they all have distinct spectra in both the VNIR and SWIR spectral ranges, allowing useful spectral analysis to be carried out in either spectral region. In particular, each pigment has strong absorption bands in the VNIR range and all behave differently with respect to each other in the SWIR region. Carmine, for example, is very transparent in the SWIR whereas Burnt Umber is opaque. In addition, Lead White, Azurite and Gypsum possess sharp and medium-strong absorption bands in the SWIR region which can be used to test the ability of the different hyperspectral systems to detect such spectral features.

The choice of a hand-made painted panel using historical pigments was made because one of the goals of the spectral imaging carried out for the project was the documentation and study of works of art and, in particular, of paintings. Natural pigments typically do not contain the kinds of abrupt absorption features seen in the wavelength standard and possess smooth continuous spectra [18]. Such a target, therefore, provides very different characteristics to more industrial materials and provides, therefore, an important test case.

**Figure 3:**
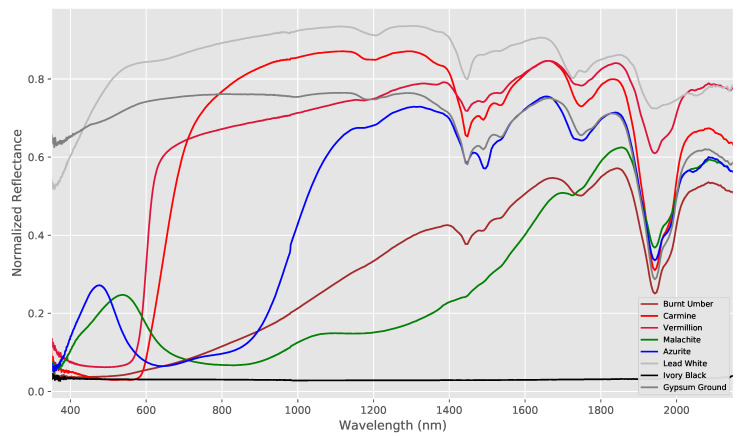
FORS reference reflectance spectra for the panel pigments.

As with the wavelength standard, a set of reference spectra was required with which to compare the acquired hyperspectral data to. These were obtained using Fiber Optic Reflectance Spectroscopy (FORS) at IFAC-CNR. FORS measurements in the UV-Visible-NIR-SWIR range were performed using two single-beam spectroanalyzers (Zeiss MCS601 Si UV-NIR & MCS611 InGaAs NIR 2.2WR) using a 0/45${}^{\circ }$ geometry. Data was calibrated and processed using the manufacturer supplied software, giving spectra with 0.8 nm and 6 nm resolutions in the VNIR and SWIR respectively resulting in a total spectral range from 350–2200 nm. The reflectance spectra of the seven pigments and ground layer are shown in Figure 3. Of course, as the hand-made target is not perfectly homogeneous, the FORS data does not necessarily provide objective spectral data. However, for the purposes of this study, it does provide a sufficiently accurate base-line from which to compare the hyperspectral data cubes.

### 2.3. Acquisition and Measurement Methodology

Each hyperspectral system acquired data with each of the two test targets. However, one of the participants (using a VNIR Headwall Hyperspec) did not provide data for the pigment panel, resulting in 8 data sets in the VNIR spectral range for the wavelength standard and only 7 data sets for the pigment panel. All 6 SWIR systems provided data for both targets.

The hyperspectral data itself was acquired and processed by each institution under the operating conditions that were standard for the institution and equipment. The acquisition parameters were all set to values considered appropriate for obtaining high quality data from the targets. The list of available parameters is provided in the Appendix B in Table A2 and the characteristics of the final data can be found in Table 1. Other parameters such as detector gain, were kept at the default level of 1 by all participants. For the illumination, all systems used similar 100 or 150 W variable Tungsten-Halogen light sources with smooth spectra that covered the whole of the spectral ranges of the cameras. However, many of the systems allow variable output and the precise illumination levels are, therefore, not known. Additionally, for the majority of systems, the acquisition integration times were not recorded by the manufacturer-supplied acquisition software and are, therefore, not available. Nor is the closely related translation stage scan speed, which was not recorded by any of the acquisition software.

After acquisition and processing carried out by the participating institutions, the resulting final calibrated spectral images were provided for analysis. The spectral data used in this paper (available as Supplementary Material) was extracted directly from these spectral images using identical protocols to ensure any variability in the spectra came from the data and not from the analytical methodology.

For both the wavelength standard and the pigment panels, the representative reflectance spectra from the acquired hyperspectral data was obtained by averaging the spectra over several hundred or thousand pixels. In this way, noise was significantly reduced and inhomogeneities averaged out.

In the case of the wavelength standard, the used pixel spectra were taken from a central circular region of size $\frac{2}{3}$ of the diameter of the target. For the pigment panel a square region within each pigment within the hyperspectral image was extracted, which was chosen to be approximately coincident to that used for physically obtaining the FORS reference spectra. The region was chosen to be, once again, $\frac{2}{3}$ of the size of the width of the pigment strip in order to allow the data to be averaged.

## 3. Results and Analysis

In this section, we will look in detail at the results obtained from the different VNIR and SWIR hyperspectral systems for both the wavelength standard and pigment panel. There are a number of ways to evaluate and quantify the hyperspectral data obtained and we will examine a range of these. These include various quantitative measures for how accurately the spectra have been reproduced, the level of noise within each data set, the geometric accuracy of the images and the fidelity of the colorimetry of each system.

### 3.1. Spectral Accuracy

The most important criteria for a hyperspectral imaging system is how accurately the spectra are reproduced. However, quantifying this is not always straightforward as there are various ways to quantify spectral accuracy and the appropriate measure of accuracy can depend on how the results will be used. For example, when using hyperspectral data for classification tasks, measures of “spectral distance” are generally used, whereas when using data for spectroscopy, factors such as the accuracy of the localization of spectral features will be more important. In this section, therefore, we will use a number of different ways to evaluate the spectral accuracy of the systems, including residual errors, distance metrics, spectral mis-alignment, spectral feature detection and how accurately the derivatives are reproduced.

#### 3.1.1. Spectral Errors

**Figure 4:**
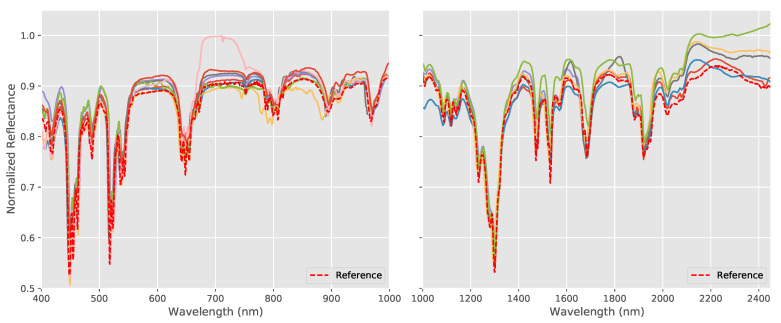
Reference and measured spectral reflectances for the wavelength standard for the visible and near infra-red (VNIR) systems (**left**) and short-wave infra-red (SWIR) systems (**right**), where the colored lines represent the individual spectra measured by each system. The systems all produced similar spectra and were able to reproduce the main spectral features in the standard. However, there are small but clear differences in the measured spectra and small spectral mis-alignments for certain systems.

The spectral responses of the different systems for the wavelength standard are shown in Figure 4 together with the reference values supplied by the manufacturer. All systems were able to produce spectra that are similar to the reference values for both the VNIR and SWIR cameras and which correctly detect the main spectral features present in the wavelength standard. However, a more detailed examination shows that there are small but clear differences in the form of the spectra and, in addition, small spectral mis-alignments in the location of the spectral features between the reference spectra and the data from the various hyperspectral imaging systems.

If we consider the responses with respect to wavelength, we see that the spectral responses are close to the reference for the majority of the spectral ranges of each system, but a number of the SWIR systems have problems beyond 2000 nm, where differences in both the shape and amplitude are evident.

The reference target contains sharp narrow peaks and troughs, which are in several cases, beyond the spectral resolution of the hyperspectral imaging systems. Each hyperspectral system has slightly different wavelength ranges, different numbers of bands with different central wavelengths and different bandwidths for each band. Therefore, in order to make meaningful quantitative comparisons, it was first necessary to resample each of our data sets to a common discretization. As the traceable laboratory reference values have a higher sampling resolution of 0.5 nm, this reference was resampled using the given center wavelengths and the given FWHM (full width half maximum) for each band for each camera. The reference spectra were essentially convolved at each center wavelength for each band with a Gaussian with the given FWHM in order to mimic the spectral and bandwidth characteristics of each of the different hyperspectral systems.

**Figure 5:**
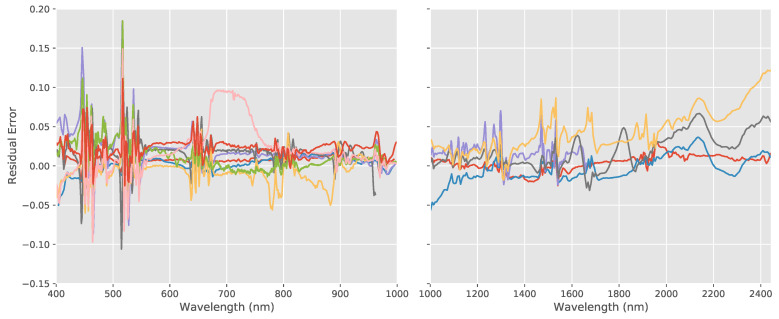
Residual errors between measured reflectance and reference spectra for wavelength standard for the VNIR (**left**) and SWIR systems (**right)** where the colored lines represent the individual errors calculated for each system. Errors are relatively small ranging from 0.05–0.18 for the VNIR systems and 0.01–0.15 for the SWIR systems.

From these resampled reference spectra, the residual error was calculated for each system by subtracting the measured spectra from the reference spectra for each band. These residual errors can be seen in Figure 5. As we can see, there are large errors at the sharp peaks and troughs, but otherwise taking the average errors and trends, we see that the error ranges are relatively small, ranging from 0.05–0.18 for the VNIR systems and 0.01–0.15 for the SWIR.

**Figure 6:**
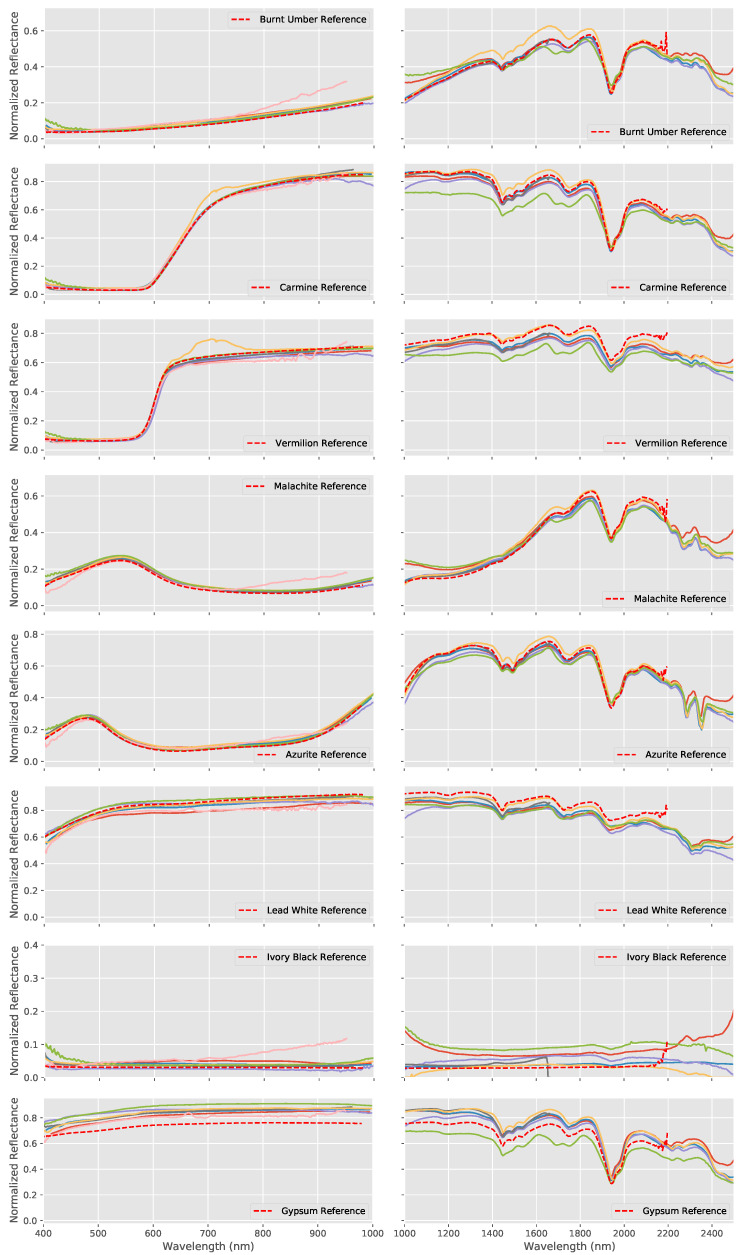
Spectral reflectances measured by each hyperspectral system (VNIR left and SWIR right) together with the FORS reference for the ensemble of pigments used on the pigment panel plus the Gypsum ground layer, where the colored lines represent the individual spectra measured by each system (note that the FORS wavelength range has a maximum of only 2200 nm in the SWIR region). The spectral shapes are all accurately reproduced, especially in the VNIR region.

The results for the pigment panel are shown in Figure 6 which shows the processed spectra measured by each hyperspectral system together with the FORS reference for the ensemble of pigments used on the pigment panel plus the Gypsum ground layer. As with the wavelength standard, both the VNIR and SWIR systems were able to broadly reproduce the overall shape and essential features of the spectra of each pigment. Although the spectra match very closely in the VNIR in most cases, there is again more variation in the SWIR spectra both in terms of spectral shape and in terms of the amplitudes of the spectra.

#### 3.1.2. Spectral Alignment

**Figure 7:**
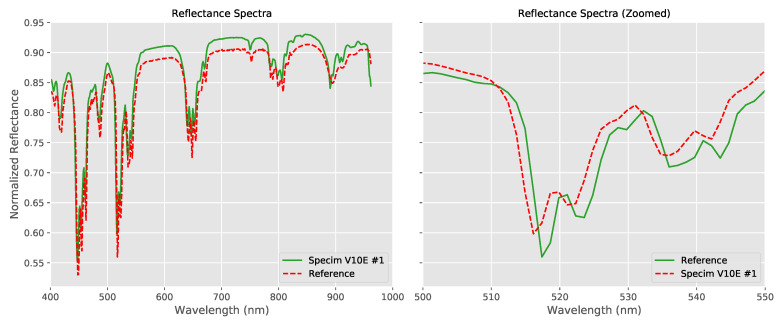
Comparison of the reflectance spectra of wavelength standard for one of the hyperspectral systems with the reference values. The zoomed view (right) shows more clearly the spectral mis-alignment.

Closer inspection of the data from Figure 4 shows that there are slight mis-alignments between the spectra acquired by the hyperspectral systems with respect to the reference. This can be seen more clearly if we take the results from a single system and Figure 7 shows the full VNIR spectra for the reference and for a single data set. The spectral mis-alignment is clearly visible in the zoomed view on the right, showing a narrower range of wavelengths.

**Figure 8:**
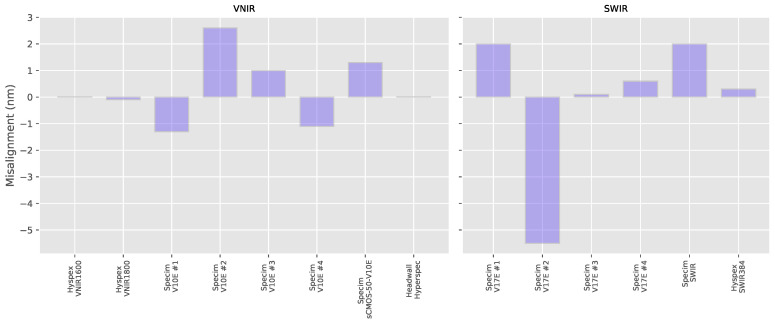
Errors in the spectral alignment for each hyperspectral system (colored lines) calculated from the data from the wavelength standard.

In order to quantify this spectral mis-alignment seen in several of the hyperspectral cameras, the derivative of the spectra was used, as the target contains a large number of narrow peaks and troughs. The zero-crossings in the derivative correspond to the top and bottoms of the peaks and troughs of the reflectance spectra and phase correlation [19] was applied to the derivative of the spectra to calculate a global offset between the resampled reference signal and the measured spectra. In Figure 8, we can see the alignment error for each system (the values are provided in the Appendix B in Table A3). The mis-alignment ranges from 0.0–2.6 nm for our different VNIR hyperspectral systems with an average mis-alignment magnitude of 0.93 ± 0.83 nm, which is less than the average distance between center wavelengths. The SWIR systems behave very similarly with error magnitudes ranging from 0.1–5.5 nm and an average mis-aligment magnitude of 1.75 ± 1.05 nm, which is again less than the average system bandwidth.

Mis-alignment can also vary with respect to wavelength and to evaluate this, the mis-alignment was calculated at different wavelengths by using small sub-sections of the spectra. However, no measurable difference was found with respect to wavelength for any of the systems.

#### 3.1.3. Distance Metrics

Hyperspectral imaging is often used for the purposes of material identification, which is usually performed through some form of classification. Classification algorithms [20] require a quantitative measure of the difference (or distance) between different spectra. In addition, they typically require the appropriate setting of threshold parameters, whose values are often dependent on the distance metric used and which can make an important difference to the classification results obtained. Given the central role played by distance metrics in classification workflows, it is useful to be able to quantify the range and variability of the spectral distances observed in real data sets.

A large number of ways exist for calculating this distance, each of which have their own particular characteristics and properties [21]. The most widely used of these distance metrics is the “Spectral Angle” used in Spectral Angle Mapper (SAM) classification. This measure is extensively used in remote sensing and is derived from the angle formed between a reference spectrum and the image spectrum of each pixel [22]. A number of other more recent distance metrics exist, including Spectral Information Divergence [23], Spectral Correlation Mapping [24], Spectral Gradient Angle [25] amongst many others. Although these metrics improve on SAM, SAM remains the most widely used distance measure thanks to its relative simplicity and integration in many hyperspectral processing software packages.

If we calculate the spectral distance between the measured reflectance spectra and the reference spectra, we can obtain a value for the accuracy of each system. Knowing how the measurement errors found in typical hyperspectral data affect distance metrics provides useful information for the setting of thresholds in classification workflows and for understanding the results and limits from classification.

**Figure 9:**
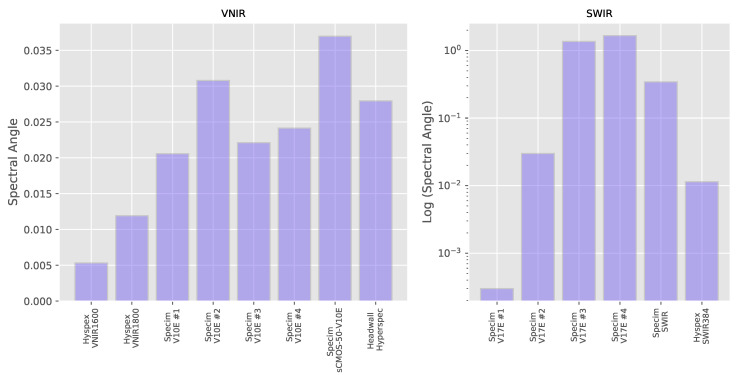
“Spectral Angle” calculated between the spectra measured by each hyperspectral system and the reference for the wavelength standard. (Note that the vertical axis of the SWIR data is a logarithmic scale due to the wide range of scores).

These errors can be seen in Figure 9, which shows the Spectral Angle distance (Equation (A1) in the Appendix A) between each measured spectra and the appropriately resampled reference for the wavelength standard. The distances are listed in the Appendix B in Table A3. The average Spectral Angle for the VNIR systems is 0.0211 for the VNIR and 0.567 for the SWIR systems.

**Figure 10:**
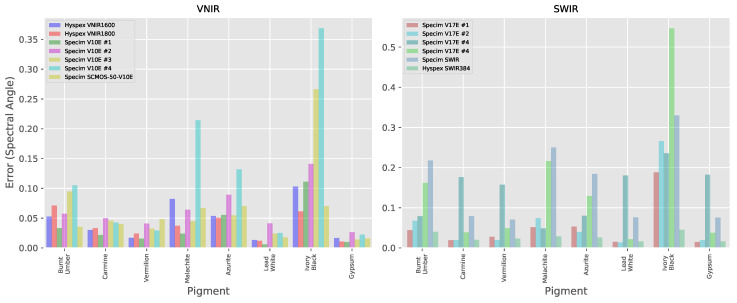
Spectral Angle error between the spectra measured by each hyperspectral system and the FORS reference for each (thickly applied) pigment.

**Figure 11:**
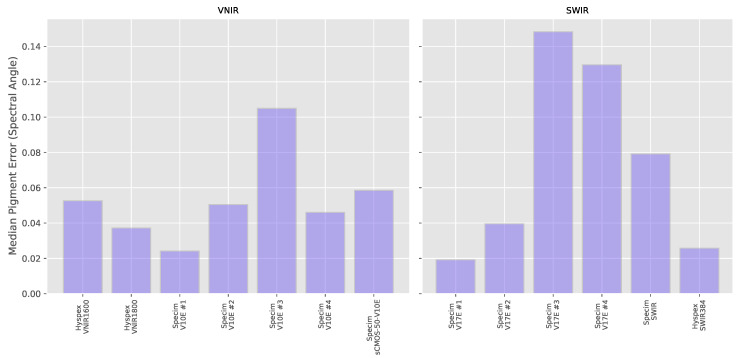
Median of the Spectral Angle errors over the ensemble of pigments and Gypsum ground layer for each hyperspectral system.

The results for each individual pigment in the pigment panel can be seen in Figure 10. The median of the result over all pigments (both thickly and thinly applied as well as for the Gypsum ground) for each individual system can be seen in Figure 11 with the distances listed in Table A3. As the FORS reference values in the SWIR region cover a smaller range of wavelengths (up to around 2200 nm) than the MCT -based systems, only the subset of the hyperspectral data that was coincident with the reference was used for calculating the Spectral Angle. The average over all pigments in the VNIR is 0.0608 ± 0.0261 and 0.0890 ± 0.0481 in the SWIR, so although the average magnitude of the errors are similar in both wavelength ranges, there is more variability in the results from the SWIR systems. If we look in more detail at the individual pigments, we note that in the VNIR region, the Spectral Angle error looks to be correlated to the average level of reflectance with the most reflective materials, Lead White and Gypsum. having lower errors than the colored pigments and the very dark pigment, Ivory Black which has a reflectivity of only around 3%, having by far the largest errors.

As we saw in Section 3.1.1, the average error is again higher in the SWIR than the VNIR systems for both targets.

#### 3.1.4. Spectral Feature Detection

**Figure 12:**
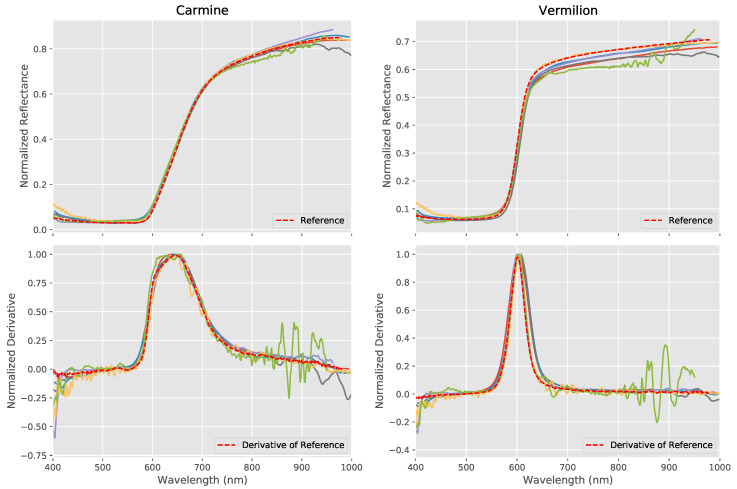
Reflectance spectra measured by each VNIR hyperspectral system along with the FORS reference for the two red pigments, Carmine (top left) and Vermilion (top right). The calculated derivatives of the respective pigment spectra from each system are shown in the bottom row. All systems were able to faithfully reproduce the reflectance spectra as well as reproduce distinct and accurate derivatives.

The top row of Figure 12 shows the spectra from the different VNIR systems for two pigments, carmine and vermilion, which have a similar red color. The samples used on the panel are in their pure form and can be easily differentiated visually in Figure 2. However, when used in a painting, such pigments will often be mixed with a white pigment, such as lead white, in order to obtain the desired shade of red. It is often, therefore, difficult to identify a pigment from its color alone and the use of distinguishing spectral features can be an important tool in pigment identification. As can be seen from their spectra, these two pigments are indeed distinguishable from the shapes of their spectra with vermilion characterized by a very sharp upward slope between around 575–625 nm, whereas carmine possesses a less steep upward slope between 600–725 nm.

A common way of studying the kind of spectral shapes seen in carmine and vermilion is through the use of derivative analysis, which can be a useful tool for the identification of pigments [26] as well as for material identification more generally [27].

The derivatives for the thickly applied patches of carmine and vermilion from each of the hyperspectral systems in the VNIR region are shown beneath their respective reflectance spectra in the bottom row of Figure 12. As certain data sets had significant levels of noise, the derivatives have been smoothed using a moving average and the derivatives normalized in order to make them comparable between systems. As we can see, the location and shape of the derivatives are faithfully reproduced by all systems.

**Figure 13:**
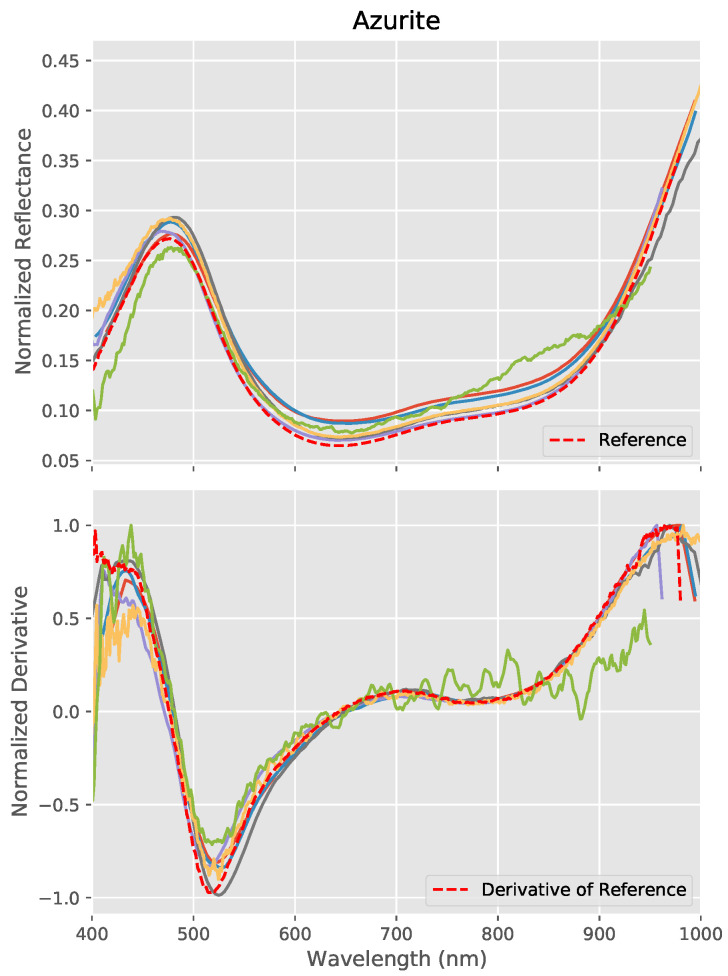
Reflectance spectra measured by each VNIR hyperspectral system (colored lines) for Azurite together with the FORS reference (**top**) and their derivatives (**bottom**). The characteristic form of the derivative is accurately reproduced, though significant levels of noise is visible in one of the results.

Azurite also possesses a very characteristic spectral shape within the VNIR region (Figure 13) with a strong peak in the blue region of the spectrum at around 480 nm which provides the pigment’s dominant color, a minima at around 645 nm and a rapid increase in reflectance after around 850 nm. Between this and the minima, there is a subtle inflection in the spectra, which gives a characteristic derivative signature. As we can see at the bottom of Figure 13, the derivative (again after application of a moving average and normalization) is accurately reproduced by all the systems, though considerable levels of noise is visible in one of the results.

To evaluate this quantitatively, the wavelength location of the peak in the blue region of the spectra and the local minima at around 645 nm were calculated for each system as well as for the FORS reference data by identifying the wavelength at which the derivative crosses zero on the Y-axis. Piece-wise linear interpolation was used to find an accurate location for the zero crossing. The results for Azurite are given in Table 2 and show that the majority of the systems were able to accurately identify the wavelength of the dominant blue peak with 4 of the systems accurate to within just 2 nm. The local minimum in the reflectance spectra proved more difficult to find accurately due to it being much broader and less well-defined. The sharp minimum in the derivative was harder to identify precisely due to noise in the derivative of the spectra, but was nonetheless correctly identified within an average of 5.2 nm.

Within the SWIR region, Azurite also has a characteristic but subtle spectral feature at around 1500 nm [26]. This feature is not easy to distinguish in Figure 6 as it is partially transparent in the infra-red and its spectra is, therefore, modulated by the Gypsum ground layer. This makes it an interesting test-case for the hyperspectral imaging systems and the “Azurite” column in Table 3 shows the error in the position of the calculated location of this feature for each system with respect to that found in the FORS reference. As we can see, despite the small size of this feature, the systems were all able to accurately locate the minima to within 3–4 nm, which is within the spectral resolution of the cameras.

A much more prominent feature can be found in the Gypsum ground layer, which possesses a strong absorption band at around 1950 nm. This can be clearly seen in Figure 6, but is beyond the spectral range of the InGaAs-based SWIR camera. Table 3 shows the errors in the wavelength location calculated for this feature for each MCT-based system and again, there is a good level of accuracy within the results with the location of the spectral feature identified with errors that are within the spectral resolution of the cameras.

### 3.2. Noise

The level of noise is an important criteria for the quality of the acquired data. The targets used in this paper have homogeneous areas which allow spectral measurements to be calculated from data averaged over several hundreds or thousands of pixels, resulting in relatively noise-free and “clean” data. However, when acquiring hyperspectral data of inhomogeneous real-world objects rather than test targets, such averaging is rarely possible and data needs to be as noise-free as possible in order to maximize its utility. Quantifying this noise is, therefore, useful and to do this we will use the most commonly used metric for measuring noise, namely the Signal to Noise Ratio (SNR).

The spectral cubes acquired from the wavelength standard were used to calculate the SNR at each spectral band with pixels were taken from a circular region with a fixed diameter of 100 pixels located at the center of the wavelength standard. For the SNR statistics to be comparable, it was necessary to use the same number of pixels from each data set. However, the resolutions of the acquired data are not identical and, thus, the collected pixels do not cover precisely the same area on the wavelength standard. Nevertheless, although not perfect, the target is sufficiently homogeneous to be able to use the data in this way to make indicative comparisons between the data.

**Figure 14:**
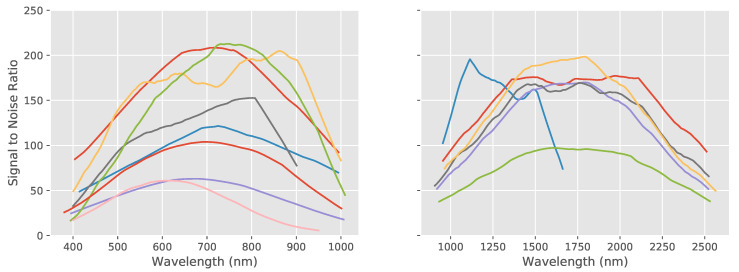
Signal to Noise Ratio (SNR) with respect to wavelength (smoothed) for each system (where the colored lines represent the SNR calculated for each system) showing how the SNR varies as a function of wavelength for each camera. As expected, SNR drops at the extremes of the wavelength range for each system. However, large differences in the amplitudes of the SNR can be seen between the acquired hyperspectral data.

The graph shown in Figure 14 shows how the SNR varies as a function of wavelength for each system.

**Figure 15:**
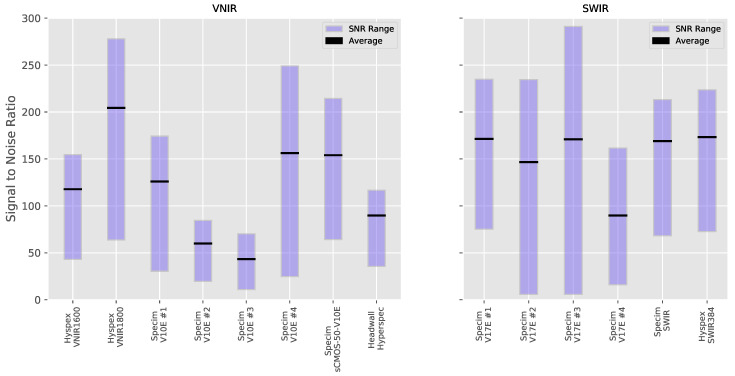
Minimum, maximum and average signal to noise ratios for each acquisition of the wavelength standard. All systems exhibit a wide range of SNR between their spectral bands and the average SNR across all wavelengths also varies considerably between systems.

Each of the spectral cubes was acquired with slightly different ranges of wavelength, as already seen in Table 1. The sensitivity of a sensor can drop dramatically at the extremes of its nominal operating range and in Figure 14 we indeed see much reduced SNR at the edges of the wavelength ranges. In order to make a meaningful quantitative comparison between the spectral cubes, a subset of the data was used which corresponds to the wavelength range common to all systems. From this subset, the SNR was calculated for each band and the max, min and average SNR over all bands were calculated. The results can be seen in Figure 15 and detailed in the Appendix B in Table A3 and we can see a wide degree of difference between the minima and maxima for each system as well as a wide spread across the systems. The minimum SNR ranges from 10.88 to 64.01 and the maximum from 70.26 to 277.88 with the average ranging from 42.77 to 203.87.

It should be noted that the SNR measures represent noise within the final processed data and not noise that is intrinsic to the system, such as detector noise etc. The differences in SNR between the systems are, therefore, not due to the hardware used, but mainly to how the acquisition was performed and the parameters used. These include factors such as the level and type of illumination, the acquisition integration time and whether noise reduction techniques such as binning or averaging have been used.

### 3.3. Spatial Accuracy

#### 3.3.1. Spatial Resolution

The hyperspectral systems tested are capable of varying levels of spatial resolution. The scans were not necessarily acquired at the maximum resolution possible for each system, but at the resolution deemed appropriate by each user given the target size, optical arrangement and the capabilities of the systems themselves. The field of view and working distances of the systems can be seen in the Appendix B Table A2 and the final spatial resolutions of the acquired data in Table 1. As we can see, there is a wide range of resolutions spanning from 3.84 to 29.2 pixels/mm with an average of 11.2 pixels/mm for the VNIR data and a narrower range of 2.26 to 3.26 pixels/mm for the MCT-based SWIR data, whose average is much lower at 3.54 pixels/mm. The single InGaAs-based SWIR system provided data at a higher resolution of 7.92 pixels/mm, though of course with less spectral range than the MCT-based systems.

#### 3.3.2. Geometric Distortion

In many applications, the spatial accuracy of the data can often be as important as the quality of the spectral data. Geometric distortion can be a common problem and there are many types of such distortion that can occur in hyperspectral imaging systems, which can make registration between VNIR and SWIR data sets or with other imaging modalities more difficult. Various optical distortions can exist that require correction and the complex multi-component optics often used in hyperspectral systems can result in complex non-linear distortions in the data that require a sensor model to correct. Data from pushbroom scan-based systems can also have distortions due to the movement and inaccuracy of the translation stages. Indeed a common problem for such scanning systems is for synchronization between the horizontal pixel resolution and the scan speed along the axis of movement. Incorrect calculation of the acquisition speed in the direction of scan movement can result in errors in the resulting aspect ratio of the data.

Many of these distortions require specialized equipment or targets to measure. The aspect ratio, however, is something that can be accurately and reliably calculated from the data acquired during the round-robin test and this was, moreover, the type of geometric distortion that was most visible and obvious in several of the data sets.

**Figure 16:**
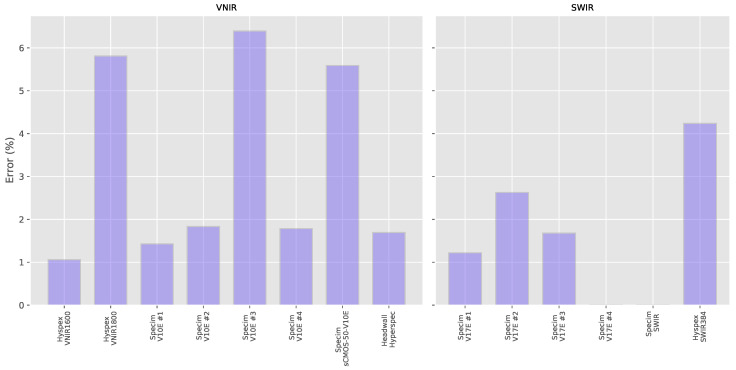
Aspect ratio errors expressed as a percentage for each system. The errors vary from zero for two of the systems to 6.39%.

To assess aspect ratio accuracy, the effective resolution was measured in both the X and Y axes and the error calculated with respect to a perfect square pixel aspect ratio of 1.0. To do this, the images from the wavelength standard were used. The wavelength standard is a perfect circle and so a Hough transform [28] was used to automatically extract the coordinates of the circle with sub-pixel accuracy and the eccentricity (if any) of the shape calculated. The results are shown in Figure 16 and the detailed results can be found in the Appendix B in Table A3. As we can see, the errors for most systems were relatively small.

Five of the eight VNIR systems had errors of less than 2% with the three others having errors of between 5% and 6%. For the SWIR systems, two systems had perfect aspect ratios, while the other four had errors ranging from 1.22% to 4.24%. These lower levels of error can be explained by the fact that the SWIR data was acquired at much lower resolution.

### 3.4. Colorimetric Analysis

Accurate colorimetry that is illuminant-independent and free of metamerism has always been an important goal for spectral imaging in fields such as cultural heritage [29,30]. It is, therefore, useful to also look at the colorimetric performance of each system. The VNIR system data was, therefore, used to calculate color coordinates in the CIELAB color space under a D65 standard daylight illuminant for each of our targets.

This was done by first determining the CIE XYZ tristimulus values through multiplying the spectral reflectance curves by the CIE standard $2^{\circ }$ observer color matching functions and by the spectral energy of the illuminant (D65 standard daylight). The values were summed and normalized for each of X, Y and Z (Equation (A2) in the Appendix A where *E* is the spectral energy and *R* is the reflectance). CIELAB coordinates were then calculated from the CIE XYZ coordinates.

The colorimetric coordinates were calculated for each target for each hyperspectral system as well as for the reference spectra. The color differences between the measured spectra and the reference were determined using CIE $\Delta E_{2000}$ [31].

Although the primary goal of the use of a wavelength standard is the assessment of spectral performance, an evaluation of the accuracy of color rendering can provide complementary information. The CIELAB color coordinates for the wavelength standard are shown in Table 4 along with the error with respect to the reference.

The colorimetric errors range from 0.36 to 5.72 $\Delta E_{2000}$ (with an average of 2.41) and the L* lightness values all closely correspond to the reference, being all within 1% of the reference. However, the a* and b* chromacity values vary far more between the data sets with there being sometimes significant errors in the b* values.

**Figure 17:**
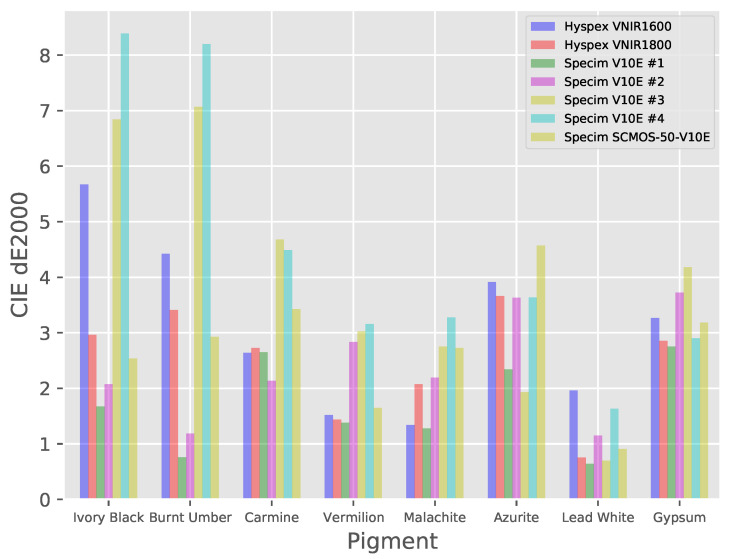
CIE $\Delta E_{2000}$ errors for panel pigments (only the thickly applied pigments are shown for clarity).

CIELAB color coordinates were also calculated for each of the different pigments from the hyperspectral data as well as for the reference spectra. The colorimetric error for each of the thickly applied pigments can been seen in Figure 17 and the average errors for each system over the ensemble of pigments are given in Table 5.

## 4. Discussion

The spectral data obtained in this study for the two test targets from the various systems and institutions show a good degree of coherence and accuracy. As can be seen in Figure 4 and Figure 6, they were all capable of distinguishing the various spectral features in the wavelength standard and were able to accurately reproduce the shapes and essential features of the pigment spectra. Using the acquired data, it was also possible to carry out reliable spectroscopic analysis and all systems were able to accurately identify sometimes subtle spectral features in both the reflectance spectra and their derivatives.

There was, nevertheless, a certain amount of variation between the results from the various hyperspectral imaging systems that were used in the study and it is useful to look in more detail at where these occur and how these errors can be reduced.

As we saw in several of the analyses, it is interesting to note that the SWIR systems had higher average levels of error than the VNIR system in the majority of the analyses and this is noticeable in Figure 4 and Figure 6, particularly at the higher end of their wavelength ranges. Particular care, therefore, clearly needs to be taken when acquiring and calibrating SWIR data.

Looking at the systems individually, no single data set was perfectly accurate, even data that had been acquired directly by the camera manufacturers. Indeed it is interesting to note that there was no consistent ranking between the various data sets when using the various measures of accuracy. In other words, systems that had the best accuracy in one category did not necessarily have the best in another.

If we consider the various measures of accuracy used, it is difficult to compare or rank the absolute magnitudes as it is the intended end-use of the data which will determine which types of accuracy are more important for a particular task. For example, for classification, accurate spectral distance measures will be critical, whereas for spectroscopy applications, spectral alignment and the ability to detect spectral features will be more important.

However, the level of variation between each of the systems for a particular measure can provide useful information and we see that the levels of SNR found within the final processed data have by far the most variability across the systems with SNR differing by up to a factor of 6 for the VNIR and 12 for the SWIR systems. The SNR measured here is the SNR found in the final processed data and is something that is affected far more by the acquisition parameters, setup and calibration than by the hardware used. This can be clearly seen in our results where there are large differences in the SNR found in the processed data even when using identical hardware. A number of straightforward improvements can be made to improve the SNR within a system [12], including tuning the acquisition parameters to the spectral characteristics of the target, the use of binning, the use of averaging or the use of equalization filters.

Other errors, such as spectral alignment errors, are difficult to correct without specialist equipment and accurate measurement, but errors in, for example, basic spatial accuracy can be improved relatively straightforwardly in many cases. For example, the aspect ratio measure used here to assess geometry only provides a very partial view of the spatial accuracy of a system, but this error is often the most visible and can encompass several types of distortion. These include optical distortions as well as errors in translation stage speed or accuracy, all of which can be corrected for.

## 5. Conclusions

A round-robin test was conducted using 14 different pushbroom hyperspectral imaging systems from 8 different institutions encompassing both the VNIR and SWIR spectral ranges. A single set of targets was used and hyperspectral data acquired using each of the imaging systems under the operating procedures, conditions and acquisition parameters that were standard for each institution and for each system. The data from each system was calibrated using the post-processing workflow that was also standard for that institution and system in order to obtain final calibrated spectral data. The aim was to compare these final data sets in order to better ascertain the reliability and comparability of hyperspectral imaging under “real-world” operating conditions where the equipment is often, to some extent, considered to be a black box. Although the study was focused on the acquisition of hyperspectral data for cultural heritage, the results will be of interest to the wider community performing laboratory-based hyperspectral imaging.

The comparison of the data shows that the majority of the systems were able to provide data that was usable and accurate enough for general use. There were, however, significant levels of variability in the data. The spectral, geometric and colorimetric accuracies were highly variable. Several of the systems measured had spectral mis-alignment errors and residual errors were common for all systems. The largest amount of variation between the systems was in terms of the levels of noise found in the processed data with SNR differing by up to a factor of 6 for the VNIR and 12 for the SWIR systems.

This variability can have an important impact on the use of such data for tasks such as material classification or for the detection of change over time. The results, moreover, highlight the importance of defining better and standardized workflows for hyperspectral imaging. By identifying the nature of these differences and by quantifying their magnitudes and variability, significant improvements can be made to the acquisition and processing pipelines. Measures, such as SNR, are highly dependent on the acquisition parameters and significant improvements are possible by the setting of more optimal parameters.

Furthermore, variability of this sort can have an impact on the behavior of metrics such as spectral distance measures. These metrics form a key component of tasks such as classification, where the choice of threshold parameters can make an important difference to the results obtained. By gaining a better understanding of the characteristics and variability of the underlying data, it will be possible to make better use of hyperspectral data for classification, change detection and other applications.

## Figures and Tables

**Table 1:** Hyperspectral cameras and the spectral and spatial characteristics of the acquired data from the round-robin test.

System	Wavelengths (nm)	Number of Bands	Average Bandwidth (nm)	Resolution (Pixels/mm)
**VNIR**				
Hyspex VNIR1600	414.70–994.01	160	3.62	29.2
Hyspex VNIR1800	404.16–994.55	186	3.17	10.7
Specim V10E #1	400.11–900.77	406	1.23	3.84
Specim V10E #2	395.58–1006.05	210	2.91	10.7
Specim V10E #3	394.80–1009.13	776	0.79	10.26
Specim V10E #4	400.65–949.95	393	1.40	4.66
Specim sCMOS-50-V10E	400.73–999.98	472	1.27	9.46
Headwall Hyperspec	382.27–1001.14	346	1.79	11.6
**SWIR**				
Specim V17E #1 (InGaAs)	954.45–1661.65	337	2.10	7.92
Specim V17E #2 (MCT)	965.18–2563.78	256	6.24	2.34
Specim V17E #3 (MCT)	919.66–2521.58	256	6.26	2.34
Specim V17E #4 (MCT)	932.20–2530.50	255	6.27	3.26
Specim SWIR (MCT)	907.16–2523.43	288	5.61	3.10
Hyspex SWIR384 (MCT)	954.98–2511.03	288	5.40	2.26

**Table 2:** Errors with respect to the FORS reference in the location of the wavelengths identified by the VNIR cameras for the blue peak, the minima in the spectral reflectance and the minima of the derivative of the pigment Azurite. (The FORS reference values for these wavelengths were 476.3, 644.1 and 518.7, respectively). The blue peak was accurately identified by the majority of the systems, while the reflectance minimum proved more difficult for the systems to accurately locate. The minimum in the derivative shows the most error, but was nonetheless correctly identified with an average error of 5.2 nm.

	Wavelength Location Error (nm)		
System	Blue Peak	Reflectance Minimum	Derivative Minimum
Hyspex VNIR1600	0.4	3.8	−4.2
Hyspex VNIR1800	1.3	9.0	−1.3
Specim V10E #1	−7.9	−0.9	−5.1
Specim V10E #2	6.0	1.2	10.4
Specim V10E #3	1.4	−1.1	6.3
Specim V10E #4	1.4	11.6	−7.2
Specim SCMOS-50-V10E	5.1	3.0	2.2
*Average Error Magnitude*	*3.4 ± 2.7*	*4.4 ± 3.9*	*5.2 ± 2.9*

**Table 3:** Error in the wavelength location with respect to the FORS reference determined by each system for two characteristic spectral features in the SWIR region for the pigments Azurite and Gypsum. For Azurite, a local minimum at 1495.5 nm and for Gypsum a strong absorption feature at 1942.5 nm (Note that the wavelength range of the InGaAs-based camera is limited to a maximum of 1661 nm and so was not able to detect the Gypsum feature).

	Error (nm)	
System	Azurite	Gypsum
Specim V17E #1	0.5	—
Specim V17E #2	−7.3	−2.7
Specim V17E #3	−1.2	4.1
Specim V17E #4	−3.3	2.8
Specim SWIR	2.7	5.5
Hyspex SWIR384	−3.7	−0.7
*Average*	*3.1 ± 2.2*	*3.2 ± 1.6*

**Table 4:** CIELAB Color Coordinates and Error for Wavelength Standard Ordered by Error.

System	L	a	b	Δ*E*_2000_
Reference	92.79	2.93	6.09	0.00
Hyspex VNIR1600	93.04	0.20	11.43	5.72
Hyspex VNIR1800	93.01	1.77	8.18	2.45
Specim V10E #1	93.64	2.66	6.55	0.36
Specim V10E #2	93.79	3.16	5.04	1.15
Specim V10E #3	93.78	2.10	5.03	1.41
Specim V10E #4	92.61	2.70	6.98	5.72
Specim SCMOS-50-V10E	93.13	3.55	7.34	1.15
Headwall Hyperspec	93.82	3.45	6.00	0.93

**Table 5:** CIE $\Delta E_{2000}$ errors averaged over all six pigments (both thin and thick layers) plus the Gypsum ground for each hyperspectral system.

System	Average Δ*E*_2000_	Std Deviation
Hyspex VNIR1600	3.09	1.42
Hyspex VNIR1800	2.48	0.93
Specim V10E #1	1.68	0.77
Specim V10E #2	2.37	0.92
Specim V10E #3	3.90	2.11
Specim V10E #4	4.46	2.34
Specim sCMOS-50-V10E	2.74	1.04

## Supplementary Materials

The spectral data used in this paper is available from https://github.com/ruven/RRT. The site includes processed spectral data of the references and the acquired system spectra from each system for both test targets. The site also features interactive graphs allowing exploration of the references and acquired spectra in more detail.

## Abbreviations

The following abbreviations are used in this manuscript:

VNIR	Visible Near Infra-Red
SWIR	Short Wave Infra-Red
BRDF	Bidirectional Reflectance Distribution Function
MCT	Mercury Cadmium Telluride
InGaAs	Indium Gallium Arsenide
CCD	Charged Couple Device
CMOS	Complementary Metal Oxide Semiconductor
FORS	Fiber Optic Reflectance Spectroscopy
UV	Ultra Violet
SAM	Spectral Angle Mapper
SNR	Signal to Noise Ratio

## Appendix A. Equations

(A1) $$SAM\left(x,y\right)=\arccos\left(\frac{\langle x,y\rangle }{{∥x∥}_{2}{∥y∥}_{2}}\right)$$

(A2) $$\begin{array}{ccc} \left[X,Y,Z\right] & = & 100*\frac{\int E_{\lambda }R_{\lambda }{\left[\bar{x},\bar{y},\bar{z}\right]}_{\lambda }d\lambda }{\int E_{\lambda }{\bar{y}}_{\lambda }d\lambda } \end{array}$$

## Appendix B. Tables

**Table A1:** Chemical composition of the pigments used in the Pigment Panel target.

Pigment	Composition
Burnt Umber	Manganese Hydroxide and Oxide with Iron Hydroxide
Carmine	Carminic acid lake
Vermilion	Mercury(II) Sulphide: HgS
Malachite	Basic copper carbonate: CuCO3·Cu(OH)_2_
Azurite	Hydrated copper carbonate: 2CuCO3·Cu(OH)_2_
Lead White	Basic lead carbonate: 2PbCO3·Pb(OH)_2_
Ivory Black	Phosphorus, calcium and magnesium with carbonate

**Table A2:** Hyperspectral cameras and the hardware and acquisition parameters used for data acquisition during the round-robin test. Note that information on the entry slit width is not available for the Hyspex systems. Also acquisition integration times are not recorded by the majority of the acquisition software and are, therefore, not provided here.

System	Detector Type	Aperture	Field of View (°)	Working Distance (cm)	Entrance Slit (μm)	Spectral Binning	Frame Averaging	Equalization Filter
**VNIR**								
Hyspex VNIR1600	CCD	f/2.5	17	30	−	2	8	True
Hyspex VNIR1800	CCD	f/2.5	17	100	−	2	20	True
Specim V10E #1	CCD	f/1.7	38	50	18	2	1	True
Specim V10E #2	CCD	f/1.7	38	50	30	4	1	False
Specim V10E #3	CCD	f/1.7	38	50	30	1	1	False
Specim V10E #4	CCD	f/1.7	38	50	30	2	1	False
Specim sCMOS	sCMOS	f/2.5	34	50	30	2	1	False
Headwall Hyperspec	sCMOS	f/2.0	34	40	25	1	1	False
**SWIR**								
Specim V17E #1	InGaAs	f/2.0	16	50	18	2	1	False
Specim V17E #2	MCT	f/2.0	16	50	30	1	1	False
Specim V17E #3	MCT	f/2.0	16	50	30	1	1	False
Specim V17E #4	MCT	f/2.0	16	50	30	1	1	False
Specim SWIR	MCT	f/2.0	16	50	30	1	1	False
Hyspex SWIR384	MCT	f/2.0	17	30	−	1	20	False

**Table A3:** Analysis data for both the VNIR and SWIR systems for the spectral mis-alignment, signal to noise statistics (mean, minimum and maximum), aspect ratio errors and Spectral Angle errors for the wavelength standard and the pigment panel (the median over the ensemble of the pigments) respectively. (Note that one of the VNIR systems did not acquire pigment panel data and, thus, the Spectral Angle error for that system and target is not available).

System	Spectral Mis-Alignment nm	SNR			Aspect Ratio Error %	SAM Error	
		Mean	Min	Max		Wavelength Standard	Pigment Panel
**VNIR**							
Hyspex VNIR1600	0.0	117.29	42.99	154.7	1.06	0.00534	0.0527
Hyspex VNIR1800	−0.1	203.87	63.62	277.88	5.81	0.0119	0.0373
Specim V10E #1	−1.3	125.46	30.31	174.32	1.43	0.0206	0.0242
Specim V10E #2	2.6	59.39	19.59	84.44	1.83	0.0307	0.0505
Specim V10E #3	1.1	155.72	24.67	249.26	1.78	0.0242	0.0460
Specim V10E #4	1.0	42.77	10.88	70.26	6.39	0.0221	0.1050
Specim sCMOS	1.3	151.46	64.01	214.5	5.59	0.0370	0.0585
Headwall Hyperspec	0.0	89.24	35.47	116.75	1.69	0.0279	—
*Average Magnitudes*	*0.93 ± 0.83*	*118.40 ± 50.04*	*36.44 ± 18.22*	*167.76 ± 70.90*	*3.20 ± 2.14*	*0.0211 ± 0.0117*	*0.0534 ± 0.0234*
**SWIR**							
Specim V17E #1	0.1	170.89	75.12	235.01	1.22	0.000301	0.0192
Specim V17E #2	0.3	170.44	5.63	291.26	2.63	0.0300	0.0396
Specim V17E #3	0.6	146.11	5.62	234.5	1.68	1.36	0.1483
Specim V17E #4	−5.5	89.32	15.91	161.61	0.0	1.66	0.1296
Specim SWIR	2.0	168.48	68.19	213.21	0.0	0.342	0.0792
Hyspex SWIR384	2.0	172.77	72.52	223.68	4.24	0.0114	0.0258
*Average Magnitudes*	*1.75 ± 1.84*	*153.00 ± 29.88*	*40.50 ± 31.70*	*226.54 ± 38.13*	*1.63 ± 1.49*	*0.567 ± 0.682*	*0.0736 ± 0.0503*
